# Clozapine for severe (“kraepelinian”) schizophrenia: Sustained improvement over 5 years

**DOI:** 10.1590/S1980-57642009DN20100014

**Published:** 2008

**Authors:** Ricardo de Oliveira-Souza, Rogério Paysano Marrocos, Jorge Moll

**Affiliations:** 1 Unidade de Neurociência Cognitiva e Comportamental, Rede LABS-D’OR, Rio de Janeiro.; 2 Hospital Universitário Gaffrée e Guinle, Universidade Federal do Estado do Rio de Janeiro.; 3 Instituto Phillippe Pinel, Rio de Janeiro.

**Keywords:** clozapine, antipsychotic, neuroleptic, kraepelinian, schizophrenia, clozapina, antipsicótico, neuroléptico, esquizofrenia, kraepeliniana

## Abstract

Clozapine has become a keystone in the treatment of schizophrenia because of its efficacy as an antipsychotic with negligible neuroleptic effects. The long-term stability of its effects, however, is poorly understood, because most studies have probed the usefulness of clozapine over a period of weeks to several months at the most. Knowing whether clozapine’s benefits are sustained over the very long-term, i.e., more than 5 years, may be critical for cost-benefit analyses.

**Objective:**

To report the results of an open study on the efficacy of clozapine over the very long-term.

**Methods:**

Thirty-three adults (26 men) with severe (kraepelinian) schizophrenia were assessed at regular intervals using a brief neuropsychiatric battery over a 5-year period.

**Results:**

A significant improvement was observed between the pre-clozapine and the first “on-clozapine” evaluation. This improvement was paralleled by a remarkable conversion of schizophrenia from “active” (mostly paranoid) into “residual” in 70% of all patients. Eight patients became functionally productive to the point of being capable of living an independent life. Roughly one-third of our cases showed no improvement.

**Conclusions:**

Clozapine is a safe and effective drug for patients with severe schizophrenia who have failed to improve on other antipsychotic drugs. Clozapine’s maximal benefit is established by the end of the first year of treatment and continues unabated for many years thereafter. Clozapine-resistant patients remain a major challenge calling for the discovery of new treatments for schizophrenia.

## Abstract (pt)

A clozapina revolucionou o tratamento da esquizofrenia em virtude de sua comprovada eficácia como antipsicótico dotado de efeitos neuroléticos mínimos. A estabilidade de seu benefício terapêutico de longo-prazo, todavia, ainda é pouco conhecida, uma vez que a maioria dos estudos comprovou sua utilidade por períodos de semanas a meses. Caso os benefícios da clozapina se sustentem por prazos maiores, i.e., por mais de 5 anos, esta informação será relevante para análises de custo-benefício.

**Objetivo:**

Relatar a eficácia da clozapina por prazos maiores em estudo aberto.

**Métodos:**

Trinta e três pacientes (26 homens) com esquizofrenia grave (“kraepeliniana”) foram examinados a intervalos regulares com uma bateria psiquiátrica breve por mais de 5 anos.

**Resultados:**

Melhora significativa foi observada entre a avaliação pré-clozapina e a primeira avaliação com clozapina, o que se acompanhou de uma notável conversão da esquizofrenia de “ativa” (constituída, na maioria, do subtipo paranóide) em “residual” em 70% dos pacientes. Oito pacientes se tornaram funcionalmente produtivos a ponto de levarem vidas independentes. Cerca de um terço dos nossos casos não melhoraram.

**Conclusões:**

A clozapina é segura e eficaz para pacientes com esquizofrenia grave que não responderam a outros antipsicóticos. Naqueles que responderam, seu benefício máximo se estabelece ao longo do primeiro ano de tratamento e assim permanece pelos anos subseqüentes. Pacientes resistentes a clozapina constituem grande incentivo para a descoberta de novos tratamentos para a esquizofrenia.

Clozapine may control psychosis in patients with schizophrenia in whom even the newer antipsychotic drugs have failed.^1^ Together with a virtual lack of neuroleptic (i.e., “extrapyramidal”) effects,^2^ clozapine’s unique profile endows it with a differential advantage over most antipsychotic drugs.^3^ Despite the large number of well designed studies conducted in the past decades that have probed the efficacy of clozapine over periods ranging from a few weeks up to a few months, few investigations have addressed the stability of the antipsychotic effect of clozapine over the long-term. In a previous article we reported our experience with the first 6 months of treatment of schizophrenia using clozapine.^4^ The results of this earlier study were in strong agreement with those involving larger series of patients. For example, one of the first studies on the time for clozapine response in a series of 50 patients with refractory schizophrenia showed that those who eventually improved, i.e., 68% of patients, did so within the first 8 weeks of treatment.^5^ In a double-blind study, the response of 240 patients treated with clozapine or with a conventional neuroleptic were compared.^6^ At the end of one year, more patients treated with clozapine had improved. The differential psychopathologic and quality of life responses for clozapine were observed, within 6 weeks and 6 months of treatment, respectively. In all, the relatively few studies published so far have endorsed the original view that clozapine is clearly valuable when other antipsychotics fail.

The extent to which the therapeutic benefits of clozapine are sustained over several years has not yet been established. The present paper reports the effects of clozapine in patients with schizophrenia who were followed for more than five years. This time period encompasses the minimum time span necessary for a categorical diagnosis of “Kraepelinian schizophrenia” and also supports the recently operationalized concept of “schizophrenia in remission”.^7^ Patients with Kraepelinian schizophrenia (KS) conform to the original prototype of “dementia præcox”,^8^, the essential characteristic of which is a persistent dependency on others for the provision of basic needs such as feeding, hygiene, shelter, financial management, and clothing.^9^ KS cuts across all DSM-IV™ subtypes of schizophrenia, but is not part of the dsmdsmdsm classification schema. Patients who are eligible for clozapine treatment usually pertain to the KS category.

## Methods

The 7 women and 26 men (plus 13 others who dropped out of clozapine treatment before the end of the first year) on whom this report is based were drawn from a larger group of 101 patients with schizophrenia diagnosed according to DSM-IV™. criteria^10^ that were eligible for a program catering to difficult-to-treat psychotic patients starting in 1995 at the Philippe Pinel Institute in Rio de Janeiro. All patients had previously been treated with several typical antipsychotics before they were switched to clozapine. Patients treated with other atypical antipsychotics before switching to clozapine were left out of the present analysis and will be the focus of a separate report. The disease of each patient was also classified according to subtype. Psychopathology was assessed with an anchored version of the Brief Psychiatric Rating Scale (BPRS).^11^ Akin to the original scale, this version has 18 items (such as suspiciousness and hallucinations) which are rated from 1 (absence of the symptom) to 7 (symptom is maximally present), allowing for separate ratings for positive (items 12 and 15), negative (items 3, 13, and 16), and conceptual disorganization (item 4) subscales. Global cognitive status and overall socio-occupational level were rated with the MMSE^12^ and the GAF scale^10^, respectively. Serial evaluations with these instruments were completed at regular intervals. For the purposes of the present study, ratings obtained at the following points were used in the statistical analyses: a baseline evaluation ("pre-clozapineh") and four evaluations after switching to clozapine ("on-clozapine") at years 1, 2, 3 and 5. Patients were also classified according to whether they had KS or were functionally productive (FP). Clozapine was the sole antipsychotic used in this series, but most patients were additionally treated with antidepressants, anxiolytics, and anticonvulsants. A history of neuroleptic use before clozapine was carefully noted down and neuroleptic doses were converted into chlorpromazine-equivalents.^13^ Blood cell counts were performed at weekly intervals in the first 6 months of clozapine use and at monthly intervals thereafter. All assessments were performed during routine and follow-up interviews by two authors (rosros and rpm ). Inter-rater agreement was high (Cohen's kappa ≥0.89) for all instruments used in the present investigation. We were also interested to ascertain whether a short battery of validated instruments could be useful in the routine assessment of inpatients and outpatients by practitioners, without the addition of extra time to a typical consultation. We surmised that the ordinal quantification of selected therapeutic targets might inform clinical judgment concerning the initiation, maintenance, and eventual withdrawal of medications.

Thirteen patients quit treatment before a year had elapsed due to low adherence to medication (often manifested as a refusal to follow prescriptions), poor environmental support (especially from relatives and spouse), intolerance to clozapine (drowsiness, hypersalivation, seizures, cataplexy), lack of efficacy, and death from unrelated (3 cases) and possibly related cause (I case of intravascular disseminated coagulation). It is noteworthy that the refusal to comply with pharmacological treatment could not be attributed to the development of extrapyramidal symptoms,^14^ since clozapine actually improved these symptoms completely or nearly so in all patients to the point of relieving drug-induced parkinsonism, truncal dystonia, and orofacial dyskinesias. Most probably, poor compliance resulted from denial of illness, an important barrier to adherence to medication in mental disorders.^15^

### Statistical methods

Results are expressed as means and standard deviations, (X±SD). Associations between categorical and continuous variables were assessed with the Chi-Square (χ^2^) and Spearman’s coefficient of correlation (rho), respectively. Comparisons between groups were evaluated with the Mann-Whitney *U* test. The significance of comparisons among means of each variable of interest was assessed using the repeated measures analyses of variance.^16^ The power (*d*) of a statistical test is considered to be good to excellent when higher than 0.80.^17^ A two-tailed 0.05 threshold of significance was adopted for all statistical tests.

## Results

The first psychotic episode had occurred around adolescence or early adulthood (17.6±4.9 years) in most patients, but clozapine was introduced much later (30.8±9.9 years). This delay was due to the unavailability of clozapine at the time that the older patients had first become psychotic and owing to the fact that most had been treated with several neuroleptics before a definite diagnosis of pharmacological resistance was established. There were more men in the group (*p*<0.001), but men and women did not statistically differ in age of illness onset (Mann-Whitney *U*=44.5, p> 0.07) or age at which they were started on clozapine (*U*=67, *p*>0.42).

Significant omnibus differences were observed for all variables of interest (Table). Except for the changes in the BPRS disorganization subscores, a pattern emerged from *post hoc* analyses that was replicated for the GAF, MMSE, as well as for total, positive and negative bprsbprsbprsbprs scores. Thus, a significant improvement was observed after the introduction of clozapine, but only between the PRE-clozapine and the first ON-clozapine evaluations. There were no statistical differences either among the second, third and fourth ON-clozapine evaluations, or between the PRE-clozapine and the second, third, and fourth ON-clozapine evaluations. These improvements were paralleled by an obvious "residualization" of the illness in most patients. Thus, 23 (≈70%) patients with active disease (paranoid=22, undifferentiated=1, disorganized=4, catatonic=6) were converted to the residual subtype during the first year of treatment (χ^2^=17.14, p<0.01). Remarkably, the vast majority of such patients comprised paranoid schizophrenics (χ^2^=44, p<0.0001). Concerning KS X FP dichotomy, 8 patients, none of whom were catatonic or disorganized before being started on clozapine, became functionally productive. No patients presented hematological complications from long-term clozapine use.

**Table:** Main results.

	Score range	PRE- clozapine	Year 1	Year 2	Year 3	Year 5	Mean score changes PRE-clozapine - Year 1
MMSE*	0-30	23±7.7	27±2.7	28±2.6	27±3.3	28±2.8	19%
BPRS Total*: Items 1-18	18-118	57±23	34±9	32±11	30±9	31±9	44%
Positive*: Items 12+15	2-14	9.7±6.0	4.0±2.1	3.9±2.5	4.2±2.8	3.8±2.4	59%
Negative: Items 3+13+16	3-21	9.6±4.7	7.8±4.0	7.0±3.7	6.1±3.7	6.8±3.8	28%
Disorganized**: Item 4	1-7	4.3±2.8	3.7±2.2	4.4±3.8	2.8±1.7	3.2±1.7	18%
GAF*	1-100	28±11	37±16	50±16	60±18	53±17	79%

* p< 0.001, d>0.99;

** p<0.05, d>0.74

Ten patients (≈30%) did not show a consistent or sustained response to even high doses of clozapine (750-900 mg/day) over a period of at least 3 months. The average daily clozapine dose in chlorpromazine equivalents did not statistically differ from the pre-clozapine daily neuroleptic dose of typical neuroleptics (800±270 vs. 785±750, p> 0.84). Serum prolactine levels were below 21 ηg/ml in all patients and had no association with clozapine dose (rho= .0.01, p>0.61) or length of treatment (rho=0.18, p>0.56).

## Discussion

Previous work by the authors^4^ and others^18,19^ has indicated that clozapine promotes a noticeable improvement in patients with severe schizophrenia by the end of 6-12 months. The present investigation further indicates that, following this initial response, an enduring period of clinical stability ensues which does not seem to appreciably change in the long-term (Figure). Such improvement was evident in the lifestyles and interest for their surroundings in most patients. Indeed, most patients might be considered “normal” by casual observers had they not ever presented symptoms of active schizophrenia. These changes are reflected in measures of overall cognition, psychopathology and socio-occupational functioning, such as those employed in the present study. Notwithstanding the sustained improvement in several spheres of life, most patients tended to remain socially detached and less inclined to engage in occupational and recreational activities than would be expected for individuals of similar age, education and social status. It is possible that patients with paranoid schizophrenia respond much better to clozapine than patients with catatonic, disorganized or undifferentiated disease. However, since patients with paranoid schizophrenia clearly outnumbered those with other subtypes in our sample, this conclusion must await validation in larger series of patients with a more balanced distribution of diagnostic subtypes.

**Figure:**
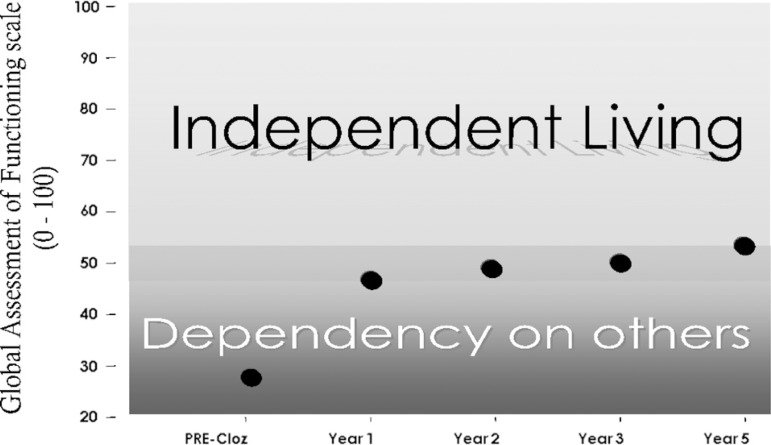
Schematic diagram of the time course of the therapeutic response to clozapine in severe (“kraepelinian”) schizophrenia. The Y-axis represents the socio-occupational response to clozapine over 5 years (ON-clozapine) taking the response to typical neuroleptics (PRE-clozapine) as a baseline. The early rapid benefit followed by stabilization over the long-term was observed for most variables of interest in the present study.

The observation that the intensity of neuroleptic treatment, as expressed in equivalents of chlorpromazine, did not differ between preprepre- and onon-clozapine epochs indicates that, contrary to the classical view,^20^ the concepts “antipsychotic” and “neuroleptic” comprise dissimilar phenomena, both at a behavioral and pharmacological level. This view is amply supported by disparate pathophysiologic mechanisms involved in the genesis of psychosis^21^ and drug-induced extrapyramidal side-effects.^22^ Typical neuroleptics produce their effects by a blockade of nigro-striatal, mesolimbic, and hypothalamo-hypophyseal post-synaptic dopamine receptors.^23^ Dopaminergic blockade at these sites is thought to be responsible for different clinically observable effects, namely, parkinsonism and other drug-induced movement disorders (nigro-striatal), amelioration of psychosis (mesolimbic), and hyperprolactinemia (hypothalamo-hypophyseal). The observation that clozapine does not induce, but rather often alleviates, drug-induced movement disorders and does not lead to hyperprolactinemia, concurs with the view that its action is rather selective for neural structures engaged by psychosis, probably mesolimbic.^24^ Strictly speaking, therefore, clozapine is an “antipsychotic”, but not a “neuroleptic”.

Our study has several limitations that may be overcome in future investigations. Because the raw data on which it is based were gathered as part of routine follow up interviews, double blind controls were not performed. Besides, the size of our sample was small and the assessment battery was not diversified to the point of allowing more specific inferences. For example, whereas there is little controversy that the improvement in negative symptoms by clozapine results from a decrease of so-called “secondary negative symptoms” (such as extrapyramidal side effects), the issue of whether “primary negative symptoms” (*i.e.*, negative symptoms which are a manifestation of schizophrenia itself) also respond to pharmacotherapy remains an open issue. Another limitation was the unavailability of blood level monitoring for clozapine. Thus, individual doses had to be tailored to each patient on the basis of clinical response alone.

In conclusion, clozapine is a safe and effective treatment in many patients with severe schizophrenia who have failed to improve on other antipsychotic drugs. This improvement is maximal by the end of the first year of treatment and continues unabated for more than five years. Clozapine-related improvement translates into cognition, psychopathology and socio-occupational performance and can be routinely measured by reliable instruments, such as the MMSE, BPRS and GAF, requiring little extra time. “Clozapine-resistant” patients, who constituted around one-third of our cases, remain a major public health concern and a pressing challenge calling for the discovery of still more efficient drugs. Following patients through numerical ratings lends structure to the clinical evaluation and often sheds light on specific problems of diagnosis and management that might go unnoticed on qualitative mental status exams. The quantifying of symptoms may greatly improve the quality of patient care even when these assessments are not intended for use in research.^25^
